# Degenerative relationships in lumbar intervertebral discs and facet joints: an MRI-based comparative study of asymptomatic individuals and patients with chronic and intermittent low back pain

**DOI:** 10.3389/fbioe.2025.1502082

**Published:** 2025-04-09

**Authors:** Hendrik Schmidt, Sandra Reitmaier, Daishui Yang, Georg Duda, Matthias Pumberger

**Affiliations:** ^1^ Julius Wolff Institute, Berlin Institute of Health at Charité – Universitätsmedizin Berlin, Berlin, Germany; ^2^ Center for Musculoskeletal Surgery, Charité – Universitätsmedizin Berlin, Berlin, Germany

**Keywords:** degeneration, facet joints, intervertebral disc, lumbar spine, morphology, MRI

## Abstract

Degeneration of intervertebral discs and facet joints are common conditions that are thought to be interrelated. This study aimed to investigate the morphological interplay between disc and facet degeneration, as well as relationships between adjacent discs and facets. This prospective study involved 712 participants (307 males, 405 females) categorized into three groups: no back pain (no-BP), intermittent (iLBP), and chronic low back pain (cLBP). The Pfirrmann classification was used to assess intervertebral disc degeneration of index and adjacent segments, while the Fujiwara classification evaluated facet joint degeneration. Spearman’s correlation coefficient analyzed relationships between degenerative changes in discs and facets. Overall, from the 712 participants 254 were with no-BP, 159 with intermittent LBP, and 299 with chronic LBP. The severity of both intervertebral disc and facet joint degeneration in the MRI sequences increased from upper to lower segments, with a significant clear directionality in differences between the uppermost and lowermost levels (p < 0.01). A strong positive correlation was observed between degenerative changes of adjacent intervertebral discs, especially in the upper and middle lumbar spine (ρ > 0.69). However, correlations between intervertebral disc and facet joint degeneration were weak in all populations studied (ρ < 0.31). The data indicate a directionality in the disease progression, with a strong correlation observed between adjacent intervertebral discs, suggesting a concurrent degenerative process. In contrast, the weak correlations between disc and facet joint degeneration imply that these structures undergo independent degenerative processes, particularly in the early stages of degeneration. Further research is essential to elucidate the underlying mechanisms and to develop precise therapeutic interventions for lumbar spine degeneration.

## Highlights

• Question

What is the relationship between the degeneration of intervertebral discs and facet joints in patients with varying levels of low back pain?

• Findings

Adjacent intervertebral discs show strong degenerative correlations, while disc and facet joint degeneration correlations are weak, especially in early stages.

• Clinical Relevance Statement

Understanding the independent and concurrent degenerative processes of discs and facets can enhance diagnostic accuracy and inform targeted therapeutic interventions for lumbar spine degeneration.

## 1 Introduction

In the healthy, non-degenerated spine, the intervertebral discs and facet joints act as an integrated unit, contributing to both load-bearing and motion control (Adams and Hutton, 1983; Ghezelbash et al., 2020; Shirazi-Adl et al., 1986; Dreischarf et al., 2016; Adams and Dolan, 2005; Arshad et al., 2019). The intervertebral discs primarily bear the majority of the axial load, distributing forces evenly across the spinal column (Adams and Roughley, 2006). This load-bearing capacity is facilitated by the unique structure of the disc, where the nucleus pulposus acts as a gel-like cushion absorbing compressive forces, while the annulus fibrosus provides tensile strength to resist deformation. Facet joints, on the other hand, play a complementary role by guiding and restricting motion, preventing excessive movement that could destabilize the spine (Shirazi-Adl and Drouin, 1987; Shirazi-Adl and Parnianpour, 1993). Importantly, the interplay between these structures ensures spinal stability and functionality (White and Panjabi, 1990). For instance, during flexion and extension, the facet joints limit excessive anterior or posterior translation, while the discs accommodate compressive and shear forces. This biomechanical synergy helps maintain spinal alignment, distributes mechanical stresses, and minimizes the risk of localized overload in individual structures.

Degeneration of intervertebral discs and facet joints are two common conditions that may lead to functional impairment (Corniola et al., 2016; Ghezelbash et al., 2020; Fujiwara et al., 2000a; Fujiwara et al., 2000b) and can cause low back pain (Rahyussalim et al., 2020; Lewinnek and Warfield, 1986; Manchikanti et al., 1999).

Intervertebral disc degeneration involves several structural changes, including a marked loss of disc height, a fibrous and dehydrated nucleus pulposus, inward and outward buckling of annulus fibrosus fibers, extensive endplate damage, and sclerosis of the subchondral bone (Adams and Roughley, 2006). Due to the greater mechanical load, intervertebral disc degeneration with advancing age primarily occurs in the lower segments of the lumbar spine (L4-L5, L5-S1) (Cheung et al., 2009). These alterations decrease the disc’s ability to absorb shock and distribute loads effectively (Ghezelbash et al., 2020). Consequently, increased mechanical stress is transferred to the facet joints (Adams and Roughley, 2006; Ghezelbash et al., 2020) and both degenerative processes are assumed to be linked to each other (Adams and Roughley, 2006; Ghezelbash et al., 2020; Fujiwara et al., 1999).

Facet joint degeneration typically manifests as cartilage erosion, formation of osteophytes, and subchondral bone changes (Fujiwara et al., 1999). Comparable to intervertebral disc degeneration, facet joint degeneration occurs more frequently in the lower segments of the lumbar spine with advancing age (Kalichman et al., 2008). These joints, which guide and limit the range of motion, experience greater loads when disc degeneration occurs, potentially accelerating their own degenerative processes (Kalichman et al., 2008).

There is a long-standing debate about possible connections between intervertebral disc and facet joint degeneration. Farfan and Sullivan (1967) and Eubanks et al. (2007) proposed that facet joint degeneration occurs first, leading to intervertebral disc degeneration due to altered load transfer. Conversely, Butler et al. (1990) and Fujiwara et al. (1999) suggested that disc degeneration precedes facet joint degeneration, potentially taking 20 years or more for facet joint osteoarthritis to develop following disc degeneration. Adding to the complexity, Suri et al. (2011) found instances of isolated degeneration in either the disc or facet joint, particularly in the elderly, raising questions about whether these processes are causally linked or occur independently.

Despite the extensive literature on adjacent segment degeneration following surgical interventions (Cannizzaro et al., 2023; Hashimoto et al., 2019), the authors are not aware of any studies that examine the likelihood of adjacent segment degeneration in the context of naturally occurring degeneration. Adjacent disc or facet degeneration may accelerate due to altered mechanical forces (Vergroesen et al., 2015), but the interplay between these structures across different lumbar levels is not fully understood. Furthermore, while MRI is commonly used to identify degenerative changes, the degree to which these imaging findings correlate with clinical symptoms like LBP is debated (Battie et al., 2009).

The primary aim of this study is to explore the relationships between MRI-verified morphological changes in intervertebral discs and facet joints across various lumbar levels. Additionally, we investigate how degenerative changes in one structure (e.g., discs) may influence adjacent structures (e.g., nearby discs or facet joints), providing a clearer picture of the degenerative processes that may contribute to LBP. By focusing on both healthy individuals and those with chronic or intermittent LBP, we seek to clarify whether these degenerative changes are specific to LBP. Understanding these relationships could improve diagnostic accuracy and inform more targeted treatment strategies for LBP, an area where current approaches often fall short.

Our study tests the following hypotheses.

i) Degeneration Clustering: Degenerative changes in intervertebral discs and facet joints will predominantly cluster in the lower lumbar spine, indicating stronger correlations between adjacent structures (disc-disc, facet-facet) in this region.
ii) Load-Dependent Degeneration: Higher mechanical loads in the lower lumbar spine will result in stronger correlations between degenerative changes in intervertebral discs and nearby facet joints, with weaker correlations in the upper lumbar spine.
iii) Independence from LBP Status: The correlation of degenerative changes identified through MRI will not be significantly influenced by the presence of low back pain, suggesting that these morphological changes occur regardless of clinical symptoms.
iv) Independence from Sex: The correlation between disc and facet joint degeneration will not be significantly influenced by sex. While some studies have highlighted sex-specific differences in lumbar disc (Miller et al., 1988; Takatalo et al., 2009; de Schepper et al., 2010; Griffith et al., 2007; Wang et al., 2011) and facet joint degeneration (Eubanks et al., 2007), these differences appear to be minimal and do not significantly alter the overall patterns of spinal degeneration.

## 2 Methods

### 2.1 Study design and population

This prospective cross-sectional study utilizes data from the ongoing “Berlin Back Study,” aimed at developing new diagnostic strategies for cLBP (https://spine.charite.de/en/spine_study/; duration: 1 January 2022 - 31 December 2025), registered with the German Clinical Trial Register (DRKS-ID: DRKS00027907). The study adheres to the ethical principles of the Helsinki Declaration and has received approval from the Ethics Committee of Charité–Universitätsmedizin Berlin (registry numbers: EA4/011/10, EA1/162/13). Each participant gave written informed consent.

Participant inclusion criteria: Caucasian men and women aged 18–64 years, provided written informed consent, and were categorized as either asymptomatic (no history of back, pelvis, or hip pain – “no-BP”) or symptomatic (either chronic low back pain – “cLBP” persisting daily for more than 3 months, or intermittent low back pain – “iLBP” lasting more than 3 months with periods of complete remission, localized in the lumbopelvic region).

Participant exclusion criteria: Participants were excluded if they were professional, competitive, or elite athletes, had acute infections, were substance abusers, pregnant, had a BMI over 28 kg/m^2^, had central or peripheral neurologic impairments (e.g., spinal cord injury, radicular symptoms, sensory deficits), had irritated, inflamed, or infected tissues in the back measurement areas, had spinal fractures, osteoporosis, tumor diseases, or bone metastases, had undergone previous spinal surgery, were on strong drug therapy (e.g., antiepileptics, long-acting antihistamines, systemic glucocorticoids, or immunosuppressive drugs), had rheumatic diseases, active systemic diseases (e.g., tuberculosis, collagenosis, multiple sclerosis, autoimmune diseases, acquired immune deficiency syndrome), had internal diseases posing potential risks during measurements (e.g., coronary heart diseases, heart insufficiency, malignant hypertension, chronic obstructive pulmonary disease), or had malpositions or anomalies of the lower extremities (e.g., knee or hip arthroplasty, arthrodesis).

### 2.2 Questionnaires

Participants provided detailed information on the location, type, duration, and intensity of their pain, along with factors that might alleviate or exacerbate it, such as physical activity (e.g., walking, sitting for extended periods), postural changes (e.g., lying down, bending), and the use of analgesics (e.g., NSAIDs, acetaminophen). Medical history, including previous diseases and surgeries, pain and general medication history, allergies, intolerances, and vaccination status, were recorded. The data were used to classify participants into the three groups: (1) no-BP: no history of back pain; (2) iLBP: intermittent low back pain lasting over 3 months with periods of complete remission; (3) cLBP: chronic low back pain persisting daily for over 3 months. The classification process involved two steps. First, participants completed a questionnaires regarding the duration, frequency, and characteristics of their LBP. This was followed by a structured interview conducted by the study physician to confirm the responses. Participants were included in the study only if their answers were consistent across both the questionnaire and the interview.

### 2.3 Spino-pelvic MRI

MRI scans of the entire lumbar spine and pelvis were performed using a Siemens Magnetom MRI machine (3.0T, Siemens AG, Germany). The sagittal plane imaging parameters included a repetition time of 682 ms and 4,800 ms, an echo time of 11 ms and 70 ms, and a slice thickness of 3 mm in T1-and T2-weighted turbo spin-echo sequences. MRIs were evaluated for intervertebral disc degeneration (IVDD) using Pfirrmann classification (Pfirrmann et al., 2001), and facet joint degeneration (FJD) using Fujiwara classification (Fujiwara et al., 1999). The Pfirrmann classification subdivides intervertebral disc degeneration into five grades, ranging from Grade I (normal) to Grade V (severely degenerated), based on disc structure, the distinction between nucleus and annulus, signal intensity, and disc height. The Fujiwara classification assesses facet joint degeneration by scoring the degree of joint space narrowing, the presence of osteophytes, and the condition of the articular cartilage. Each joint is assigned a score from 1 to 4, where 1 indicates no degeneration and 4 indicates severe degeneration. All parameters were independently evaluated by two experienced orthopedic physicians and one radiologist, each blinded to the others’ results. The final values were obtained by calculating the median of their individual assessments, ensuring an unbiased and robust measure of the parameters.

### 2.4 MRI reliability tests

**FIGURE 1:**
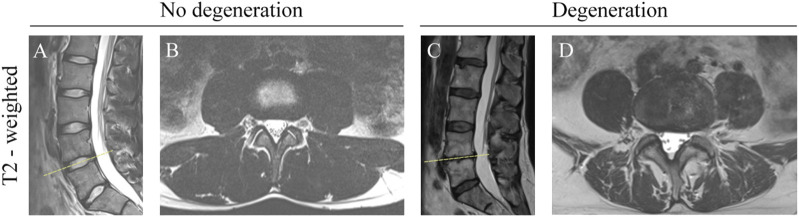
Morphometric Changes in the Lumbar Spine on T2-Weighted MRI. **(A)** Sagittal view: Non-degenerated discs showing hyperintense signal and normal height. **(B)** Transverse view: Non-degenerated facet joints with normal joint space and no osteophytes. **(C)** Sagittal view: Severe disc degeneration at L4/L5, indicated by hypointense signal and decreased disc height. **(D)** Transverse view: Facet joint degeneration at L4/L5, with obliterated joint space and osteophyte formation.

To ensure the robustness of the MRI-based assessments, inter-rater reliability was assessed (Figure 1). The reliability for IVDD assessments was substantial (Conger’s Kappa = 0.72, Gwet’s AC2 = 0.91), and for FJD assessments, it ranged from moderate to substantial (Conger’s Kappa = 0.65, Gwet’s AC2 = 0.77), demonstrating consistent evaluation across raters. Additionally, a test-retest evaluation was performed on 40 randomly selected MRI scans. Two of the three observers reanalyzed these scans 2 weeks apart, yielding an intraclass correlation coefficient of 0.89, which indicates high reliability.

### 2.5 Statistics

Spearman’s correlation coefficient was employed to assess the relationship between IVDD and adjacent IVDD, IVDD and FJD as well as FJD and adjacent FJD. The following interpretation of correlation coefficients was used: a coefficient between ±0.8 and ±0.99 indicates a very strong monotonic relationship, a coefficient between ±0.6 and ±0.79 indicates a strong monotonic relationship, coefficients in the range of ±0.4 to ±0.59 indicate a moderate monotonic relationship, coefficients in the range of ±0.2 to ±0.39 indicate a weak monotonic relationship, coefficients between ±0.1 to ±0.19 suggest a very weak monotonic relationship, and a coefficient around ±0 indicates no monotonic relationship between the two variables. The chi-square test and ANOVA were conducted to analyze categorical and continuous data, respectively.

## 3 Results

### 3.1 Study population

The study included 712 participants (307 males, 405 females) which distributed across the three groups as follows: 254 (110 males, 144 females) no-BP, 159 (67 males, 92 females) iLBP, and 299 (130 males, 169 females) cLBP (Table 1). Both iLBP and cLBP groups had a significantly higher average age compared to the no-BP group (p = 0.009). No significant differences were observed among the groups regarding sex, BMI, and smoking status. The frequency of alcohol intake was higher in the cLBP group compared to the no-BP group (p = 0.057) and the intermittent iLBP group (p = 0.038).

**TABLE 1:** Characteristics and demographics between no back pain (no-BP), intermittent low back pain (iLBP), and chronic low back pain (cLBP).

	no-BP	iLBP	cLBP	*p* - value
No. of subject	254	159	299	
Sex (male/female)	110/144	67/92	130/169	0.960
Age (median and range years)	39.0 (29.0–52.0)	41.0 (32.0–54.0)	44.0 (34.0–53.0)	0.009
BMI (kg/m^2)	23.0 (21.4–25.0)	23.5 (21.2–25.2)	23.4 (21.6–25.3)	0.500
Smoking (yes/no)	24/230	13/146	34/265	0.522
Alcohol frequency				
≤ once per week	195	125	208	<0.001^a^
> once per week	59	34	91	

### 3.2 Distribution of IVDD and FJD

**FIGURE 2:**
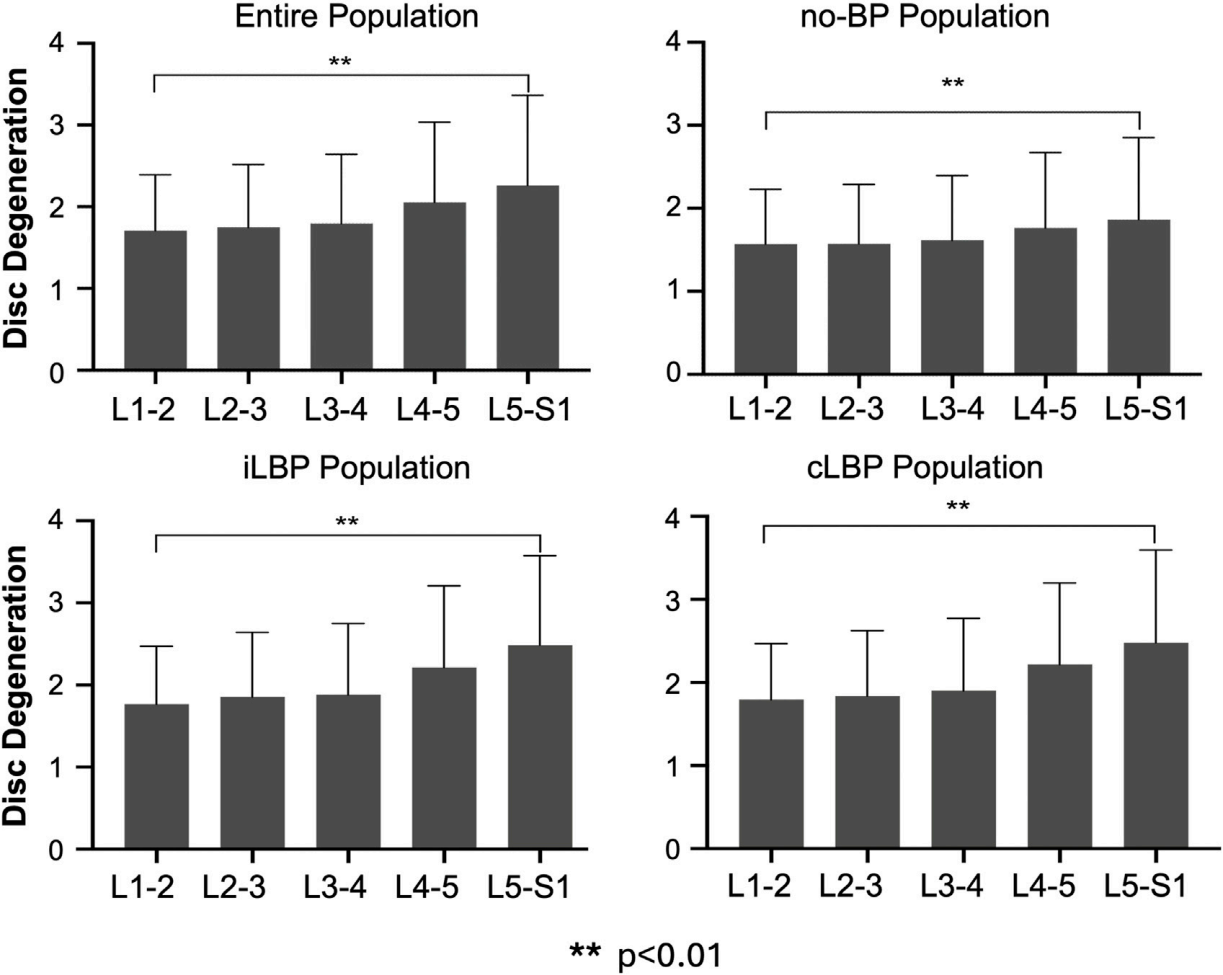
Segmental distribution of intervertebral disc degeneration in entire study population as well as subgroups with no back pain (no-BP), intermittent low back pain (iLBP), and chronic low back pain (cLBP).

**FIGURE 3:**
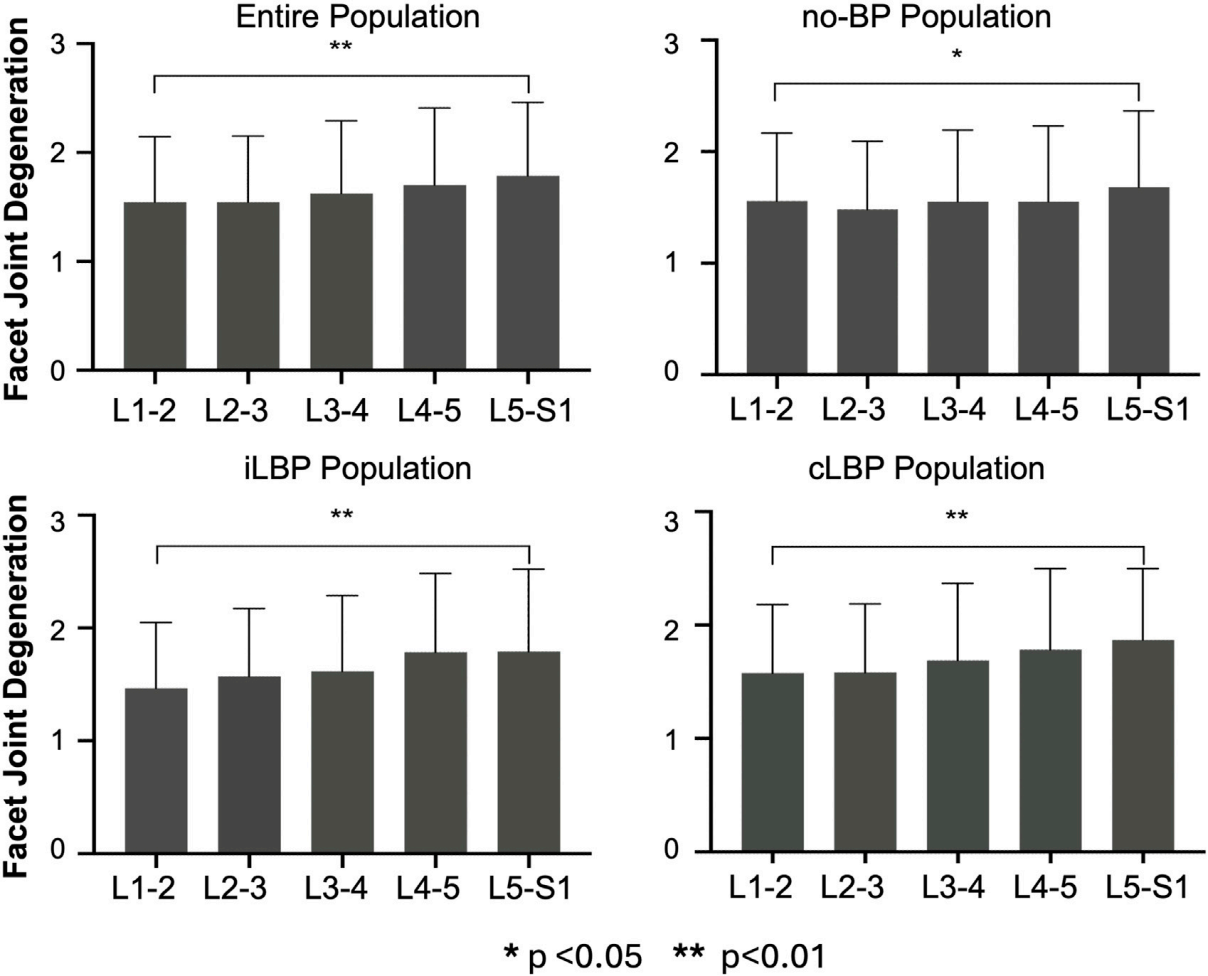
Segmental distribution of Facet joint degeneration in entire study population as well as subgroups with no back pain (no-BP), intermittent low back pain (iLBP), and chronic low back pain (cLBP).

For the entire population as well as all subgroups, the severity of IVDD increased continuously from upper (L1-L2) to lower (L5-S1) segments (Figure 2). However, significant differences were observed only between the uppermost and lowermost levels of the IVDs (p < 0.01). Similar behavior was observed for FJD in both the overall group and all subgroups, characterized by a continuous increase in degeneration from upper to lower segments (Figure 3). Significant differences were also found only between the uppermost L1-L2 and the lowest L5/S1 segments (p < 0.01).

### 3.3 Correlation between IVDD and FJD

**FIGURE 4:**
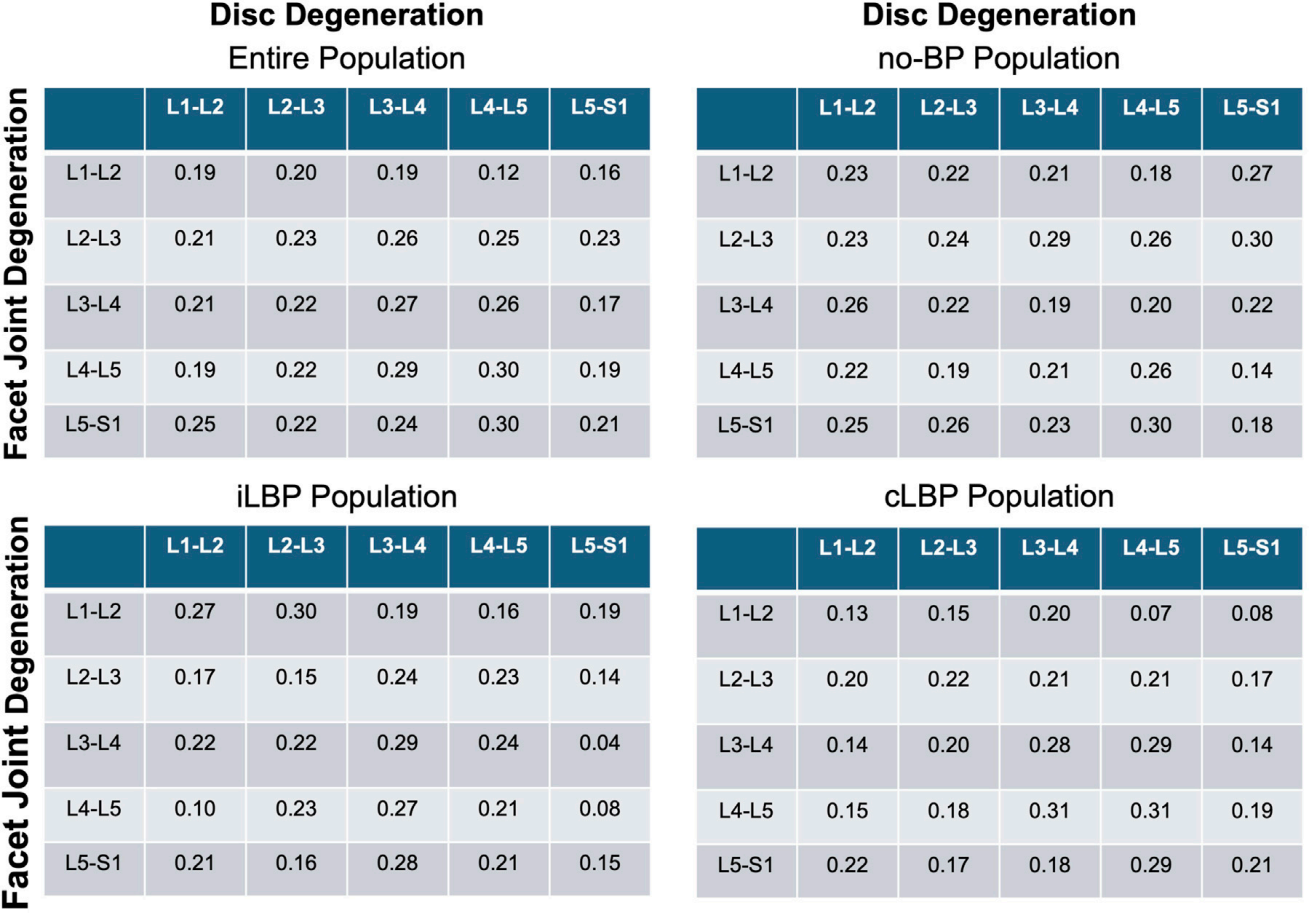
Correlation matrices of disc and facet joint degeneration. Values indicate Spearman’ correlation coefficients in each spinal segment. no-BP: no back pain, iLBP: intermittent low back pain, cLBP: chronic low back pain.

Spearman’s correlation analyses revealed a very weak to weak positive correlation between IVDD and FJD (p < 0.001) (Figure 4). This applies to all four study populations studied.

### 3.4 Degeneration patterns in adjacent discs

**FIGURE 5:**
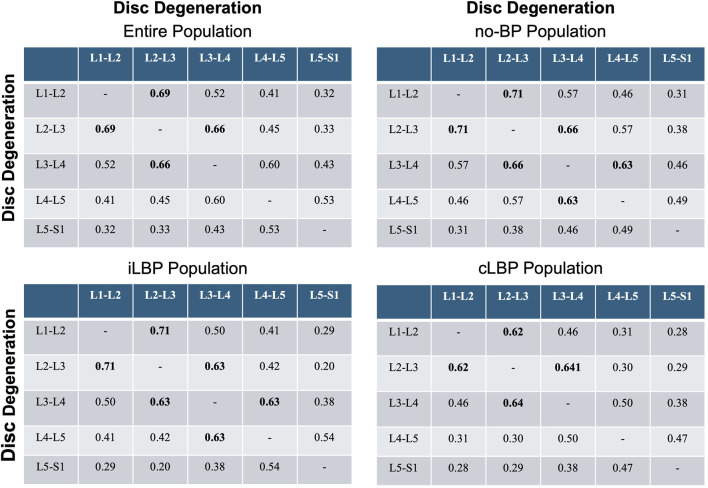
Correlation matrices of disc and adjacent disc degeneration. Values indicate Spearman’ correlation coefficients in each spinal segment. Bold values indicate strong monotonic relationships. no-BP: no back pain, iLBP: intermittent low back pain, cLBP: chronic low back pain.

Strong positive correlations (p < 0.001) was observed between IVDD and the degeneration of adjacent discs at the lumbar segments L1-L2, L2-L3, and L3-L4 (Figure 5). For the lower lumbar segments, only a moderate correlation was found. The correlation steadily decreased as the distances between the discs increased. As an example, the correlation between L1-L2 and L2-L3 was 0.69 in the entire population, decreasing to 0.52 between L1-L2 and L3-L4, and further to 0.41 between L1-L2 and L4-L5. This phenomenon was observed in all four populations.

### 3.5 Degeneration patterns in adjacent facet joints

**FIGURE 6:**
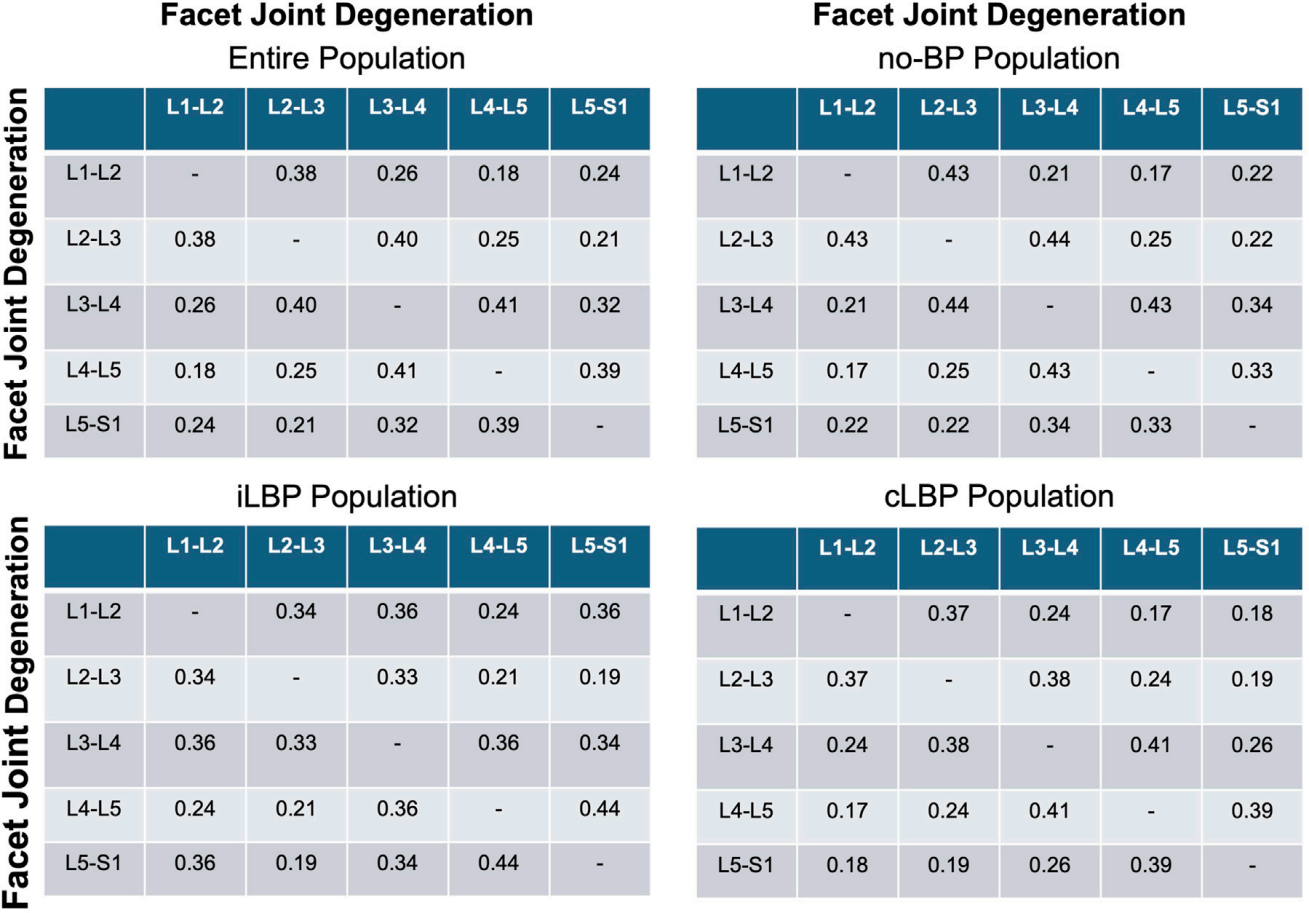
Correlation matrices of facet and adjacent facet degeneration. Values indicate Spearman’ correlation coefficients in each spinal segment. no-BP: no back pain, iLBP: intermittent low back pain, cLBP: chronic low back pain.

Very weak to weak positive correlations were found for FJD and the degeneration of adjacent FJs (p < 0.001) (Figure 6) for all four study populations.

### 3.6 Effect of different dimensions of pain

**FIGURE 7:**
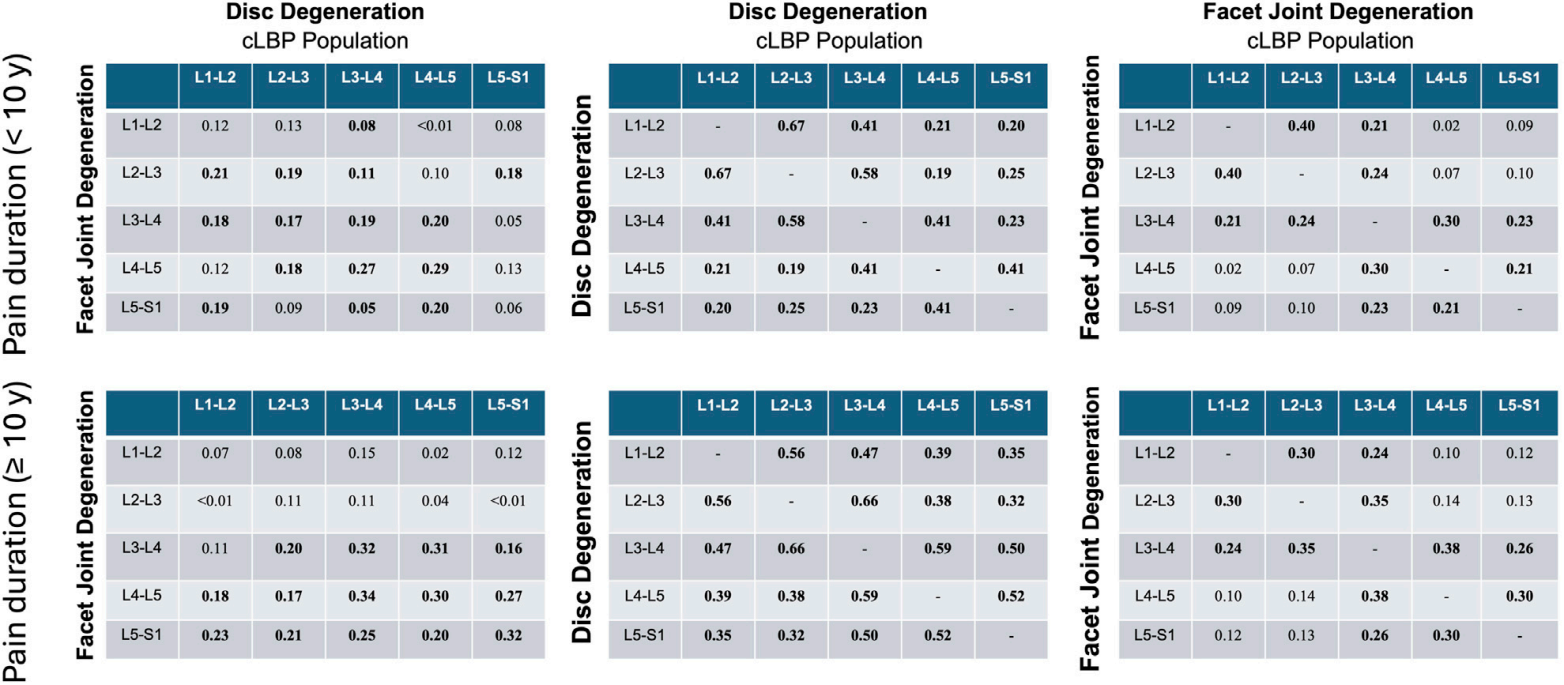
Correlation matrices for disc and facet joint degeneration, disc and adjacent disc degeneration, and facet and adjacent facet degeneration were analyzed for pain duration groups of <10 years and ≥10 years. Values indicate Spearman’ correlation coefficients in each spinal segment. cLBP, chronic low back pain.

These analyses revealed that pain intensity had negligible effects on individual correlations. However, pain duration exceeding 10 years showed significantly higher correlations between IVDD and the degeneration of adjacent discs compared to the group with a pain duration of less than 10 years, with a mean correlation of 0.12 (range: −0.11–0.27) (Figure 7).

### 3.7 Effect of sex

Sex did not have a substantial influence on the identified correlations between IVDD and FJD, IVDD and adjacent IVDD, or FJD and adjacent FJD. Maximum correlation coefficients up to 0.71 were observed between IVDD and adjacent IVDD for both sexes, indicating that sex had no significant impact on these relationships. The corresponding figures illustrating these results are provided in the Supplementary Material (Supplementary Figures S1–S6).

## 4 Discussion

The study aimed to investigate the morphological relationships between intervertebral disc and facet joint degeneration in the lumbar spine, as well as the degeneration patterns between adjacent discs and adjacent facets. Both degenerative processes are clinically treated distinct but frequently associated as coupled degenerative processes and associated with mechanical loading leading to their degeneration. Using MRI data from individuals that either suffer from no or low back pain (noBP, iLBP and cLBP), established degeneration scoring systems (Pfirrmann et al., 2001; Fujiwara et al., 1999), and Pfirrmann correlation analysis, the study provides valuable insights into the complex interplay between these spinal structures with regards to degenerative patterns in the upper lumbar spine and adjacent segment disease.

### 4.1 Hypothesis 1: degeneration clustering

Our first hypothesis posited that degenerative changes in IVDs and FJs would cluster predominantly in the lower lumbar spine, where mechanical loads are highest, leading to a strong correlation between the degeneration of adjacent discs and adjacent facets. However, the results revealed predominantly weak positive correlation between IVDD and FJD across all investigated populations, indicating that these structures may follow largely independent degenerative pathways, possibly reflecting the multifaceted nature of spinal degeneration influenced by genetic predisposition, environmental factors, and biomechanical alterations (Adams and Roughley, 2006; Suri et al., 2011). Our findings challenge the hypotheses proposed by Farfan and Sullivan (1967), Eubanks et al. (2007), who suggested that FJD precedes IVDD, and also the opposing view of Butler et al., (1990) and Fujiwara et al. (1999), who suggested that IVDD precedes FJD. Instead, our data align with studies such as those by Suri et al. (2011) and Ferguson and Steffen (2003), which propose that disc and facet joint degeneration may progress independently but are influenced by shared risk factors like mechanical loading and aging. These findings suggest that while there may be concurrent degeneration, the mechanisms behind each are distinct and may occur in parallel, without necessarily being causally linked.

### 4.2 Hypothesis 2: load-dependent degeneration

Our second hypothesis suggested that degeneration in one intervertebral disc would lead to increased degeneration in adjacent discs, especially in the lower lumbar spine where mechanical loads are highest. Contrary to this, our findings revealed that the most significant correlations in the degeneration of adjacent discs occurred in the upper lumbar spine (L1-L2, L2-L3, L3-L4), rather than the lower spine. This finding suggests that while altered load distribution and mechanical stress are important contributors to disc degeneration, the upper lumbar spine may be more vulnerable to the propagation of degenerative changes due to unique anatomical and functional characteristics. These include differences in segmental movement patterns and spinal alignment, as previously highlighted by Dreischarf et al. (2014). While earlier studies have emphasized that degeneration often initiates in the lower lumbar spine, particularly in the heavily loaded L4/L5 and L5/S1 segments (Fujiwara et al., 1999; Ferguson and Steffen, 2003), our results highlight the need to consider regional variability in the progression of degenerative changes. It is possible that degeneration in the lower lumbar spine creates biomechanical compensations that indirectly influence the upper lumbar segments, making them more susceptible to correlated degeneration. Further biomechanical and longitudinal studies are required to unravel these complex regional interactions and to clarify the mechanisms driving the observed patterns of degeneration in the lumbar spine.

We found weak positive correlations between the degeneration of adjacent facet joints, similar to the patterns observed in adjacent discs. However, these correlations were weaker compared to those seen with adjacent discs. This may be attributed to the more localized mechanical function of facet joints, which primarily guide and limit motion rather than bearing loads like intervertebral discs. The presence of weak correlations suggests some degree of interdependence, possibly mediated through altered spinal mechanics and compensatory changes in adjacent joints. These findings align with the idea that, while adjacent joint degeneration occurs, it may not be as pronounced or directly related as that seen in discs. Interestingly, a recently published study demonstrated a close association between facet joint orientation and facet joint tropism with disc degeneration (Eksi et al., 2024). According to the study, facet joint orientation and tropism not only affect the disc at the corresponding level but also at adjacent levels. This finding suggests a functional connection between these two structures, highlighting that while there is a close interaction, the degeneration of facet joints and intervertebral discs is not directly associated. These results further emphasize the complex, multifactorial nature of spinal degeneration, where structural changes in one component may not necessarily predict or directly influence changes in adjacent structures.

### 4.3 Hypothesis 3: independence from LBP status

The relationship between degenerative changes in the spine and cLBP is complex and multifactorial. Although spinal degeneration, including changes in discs and facet joints, is frequently associated with LBP, the correlation between these changes and pain is not always straightforward. Previous studies (e.g., Battie and Videman, 2006; Luoma et al., 2000) have demonstrated that some individuals with significant degenerative changes may remain asymptomatic, while others with minimal degeneration can experience severe pain. In our study, we further explored this relationship by analyzing the impact of pain intensity and pain duration on the correlation between degenerative changes and clinical symptoms. We found that pain intensity had a negligible effect on individual correlations. However, pain duration exceeding 10 years was associated with significantly higher correlations between disc and adjacent disc degeneration compared to a pain duration of less than 10 years. These results suggest that prolonged exposure to chronic pain may strengthen the relationship between degenerative disc changes and clinical symptoms. This underscores the importance of considering pain chronicity when examining the clinical implications of spinal degeneration.

### 4.4 Hypothesis 4: independence from sex

In line with our hypothesis, our study demonstrated that sex did not have a significant influence on the correlations between IVDD and FJD, nor on the relationships between adjacent discs and adjacent facet joints. Both males and females exhibited similar degenerative patterns, with the highest correlation coefficients for adjacent intervertebral discs reaching up to 0.71. These findings imply that sex is not a determining factor in the progression of spinal degeneration, suggesting that other factors—such as age, mechanical loading, genetic predisposition, or lifestyle—play a more pivotal role in the onset and development of degenerative changes in the spine.

The absence of a sex-based difference in degeneration patterns aligns with existing literature. Studies by Battie et al. (2004), and Videman et al. (2003) have demonstrated that spinal degeneration occurs similarly in men and women, with age and mechanical stress serving as the primary influencing factors. While certain studies (Miller et al., 1988; Takatalo et al., 2009; de Schepper et al., 2010; Griffith et al., 2007; Wang et al., 2011; Eubanks et al., 2007) have reported minor sex-based variations, these differences appear to have limited impact on the overall progression of spinal degeneration.

### 4.5 Clinical implications

The weak correlation between IVD and FJ degeneration highlights the need for comprehensive diagnostic approaches that consider both structures independently, rather than presuming a direct causal relationship in the early stages of disease. Conversely, the strong correlations observed in adjacent disc degeneration highlight the critical importance of early intervention in disc pathology to potentially prevent the progression of degeneration to neighboring discs. For facet joint degeneration, the study suggests that while adjacent joint involvement is less pronounced, it still warrants attention in the overall management of spinal health (Butler et al., 1990; Eubanks et al., 2007).

Our findings underscore the importance of distinguishing between intervertebral disc and facet joint degeneration when developing treatment strategies. While disc degeneration is primarily associated with load-bearing, facet joint degeneration is more related to spinal motion control. As a result, clinical interventions should be tailored based on the specific degenerative process, with approaches like spinal fusion for disc degeneration and facet joint injections or ablation for facet joint degeneration being more effective.

### 4.6 Future directions

Future research should explore the underlying mechanisms driving the observed correlations, particularly the factors influencing the propagation of degenerative changes. Longitudinal studies could provide more definitive insights into the temporal sequence of degeneration between discs and facet joints. Additionally, biomechanical modeling and *in vivo* studies could elucidate the specific load transfer mechanisms contributing to the spread of degeneration. Improved imaging techniques and the integration of genetic and environmental data will further enhance our understanding of spinal degeneration (Farfan and Sullivan, 1967; Battie and Videman, 2006).

A potential area for future research is the role of the paraspinal muscles in the degenerative processes of the lumbar spine. While our study focused on the segmental analysis of intervertebral disc and facet joint degeneration, acknowledging the interaction between these structures, the contribution of paraspinal muscle morphology and function warrants further investigation. Recent studies suggest that factors such as fatty infiltration and muscle atrophy in paraspinal muscles may influence or even exacerbate spinal degeneration (Ozcan-Eksi et al., 2023). Incorporating paraspinal muscle evaluation into future analyses could provide a more comprehensive understanding of lumbar spine pathology and offer additional insights into the biomechanical and structural factors underlying degenerative changes.

### 4.7 Study limitations

Our study has several limitations that should be considered when interpreting the findings. First, the cross-sectional design prevents us from establishing causal relationships or inferring temporal progression, which may affect the generalizability of the results. Additionally, the community-based nature of our research included fewer participants with severe back pain or significant physical functional impairments, potentially limiting the representation of this subgroup. While we made extensive efforts to account for various confounding variables, residual bias from unmeasured confounders cannot be entirely ruled out.

Furthermore, the study cohorts predominantly consisted of individuals of European ancestry, which restricts the applicability of our findings to other ethnic populations. Regarding imaging, the MRI data provided valuable insights into degenerative changes; however, limitations such as image resolution, the qualitative nature of certain assessments, and the exclusion of subtle subclinical changes may have influenced the interpretation of the results. These constraints underscore the need for caution when generalizing our findings and highlight the importance of future research incorporating advanced imaging modalities and a more diverse participant population.

We did not perform a formal power analysis prior to the study due to its exploratory nature and the constraints of the available datasets. However, we conducted a *post hoc* power analysis to assess whether the sample size was sufficient to detect correlations within the study. With a sample size of 712 participants, assuming typical Type I and Type II error probabilities of 5% and 20%, respectively (i.e., a significance level of 0.05 and a power of 0.80), the minimum detectable correlation was approximately r = 0.10 in absolute terms. This indicates that the sample size was large enough to detect even weak correlations, ensuring that no meaningful effect or relationship was overlooked—a key objective of exploratory research.

For moderate effect sizes (r = 0.3), the study achieved a power of approximately 0.99, confirming its ability to reliably identify stronger relationships between intervertebral disc degeneration and facet joint degeneration, as well as between these degenerative changes and adjacent structures. For larger effect sizes (r = 0.5), the power would be even higher. These findings highlight the robustness of the study design, demonstrating that the sample size was more than adequate to detect significant associations, fulfilling the exploratory aim of identifying potential effects and relationships.

Moreover, we did not control for the effects of age, sex, and other demographic factors. However, our preliminary analysis, which was stratified by sex, indicated that sex did not significantly impact the observed correlations. The influence of age and other demographic variables warrants further investigation. Future research could utilize matched designs or stratified analyses to address these factors more comprehensively, offering deeper insights into their potential roles in lumbar spine degeneration.

## 5 Conclusion

This study underscores the complex morphological relationships between IVD and FJ degeneration and highlights the interconnected nature of spinal structures. While the correlations are generally weak, the findings reinforce the need for a holistic approach in diagnosing and treating lumbar spine degeneration. Early detection and targeted interventions could mitigate the progression of degenerative changes, ultimately improving patient outcomes and quality of life.

## Data Availability

The original contributions presented in the study are included in the article/Supplementary Material, further inquiries can be directed to the corresponding authors.
