# Supplementary figures and images for: Degenerative relationships in lumbar intervertebral discs and facet joints: an MRI-based comparative study of asymptomatic individuals and patients with chronic and intermittent low back pain
Source: Front Bioeng Biotechnol. 2025 Apr 9;13:1502082. doi: 10.3389/fbioe.2025.1502082 (PMC12014670; doi:10.3389/fbioe.2025.1502082)

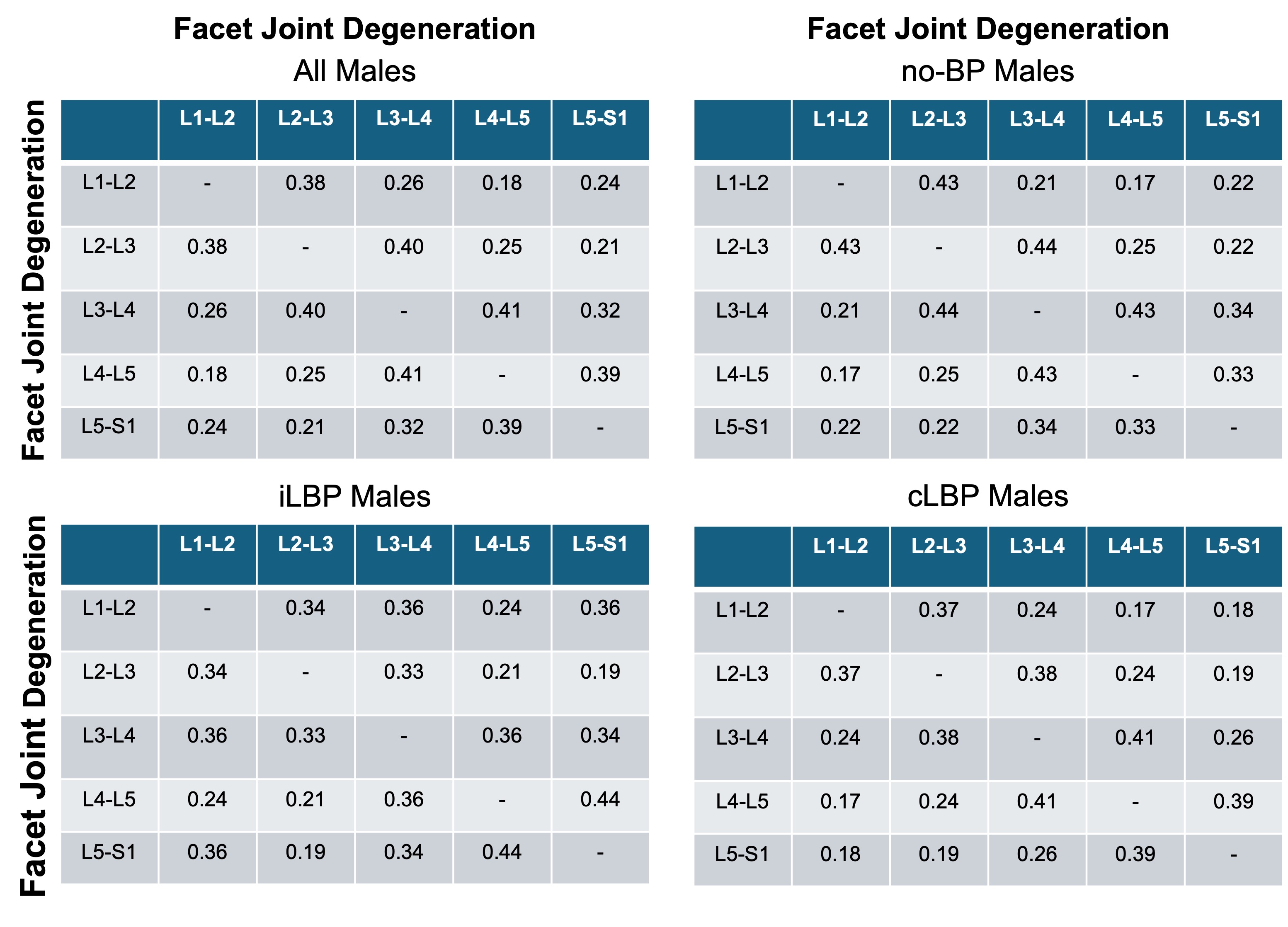

Supplement: Supplementary file 1 [file Image3.jpeg]

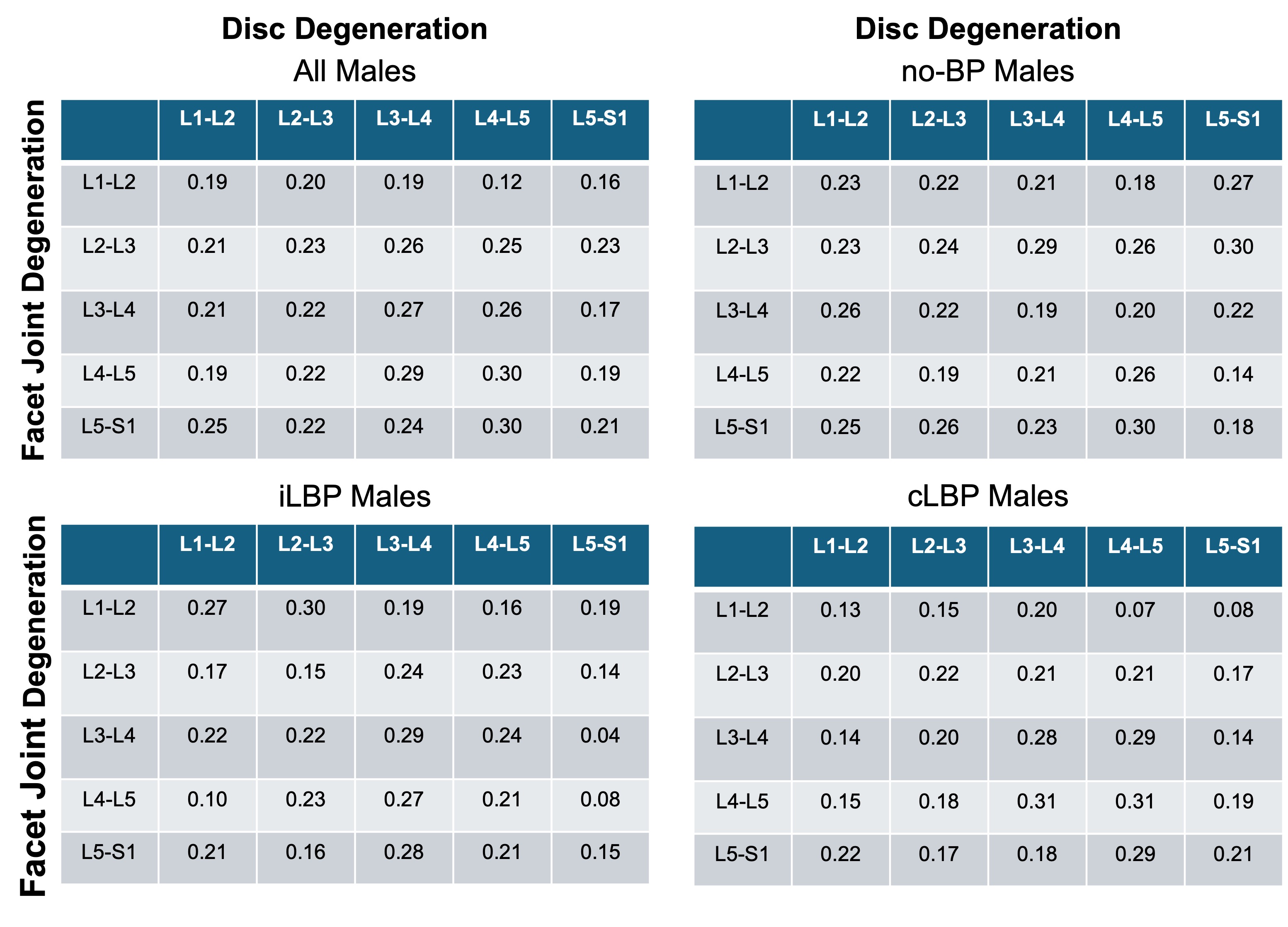

Supplement: Supplementary file 2 [file Image1.jpeg]

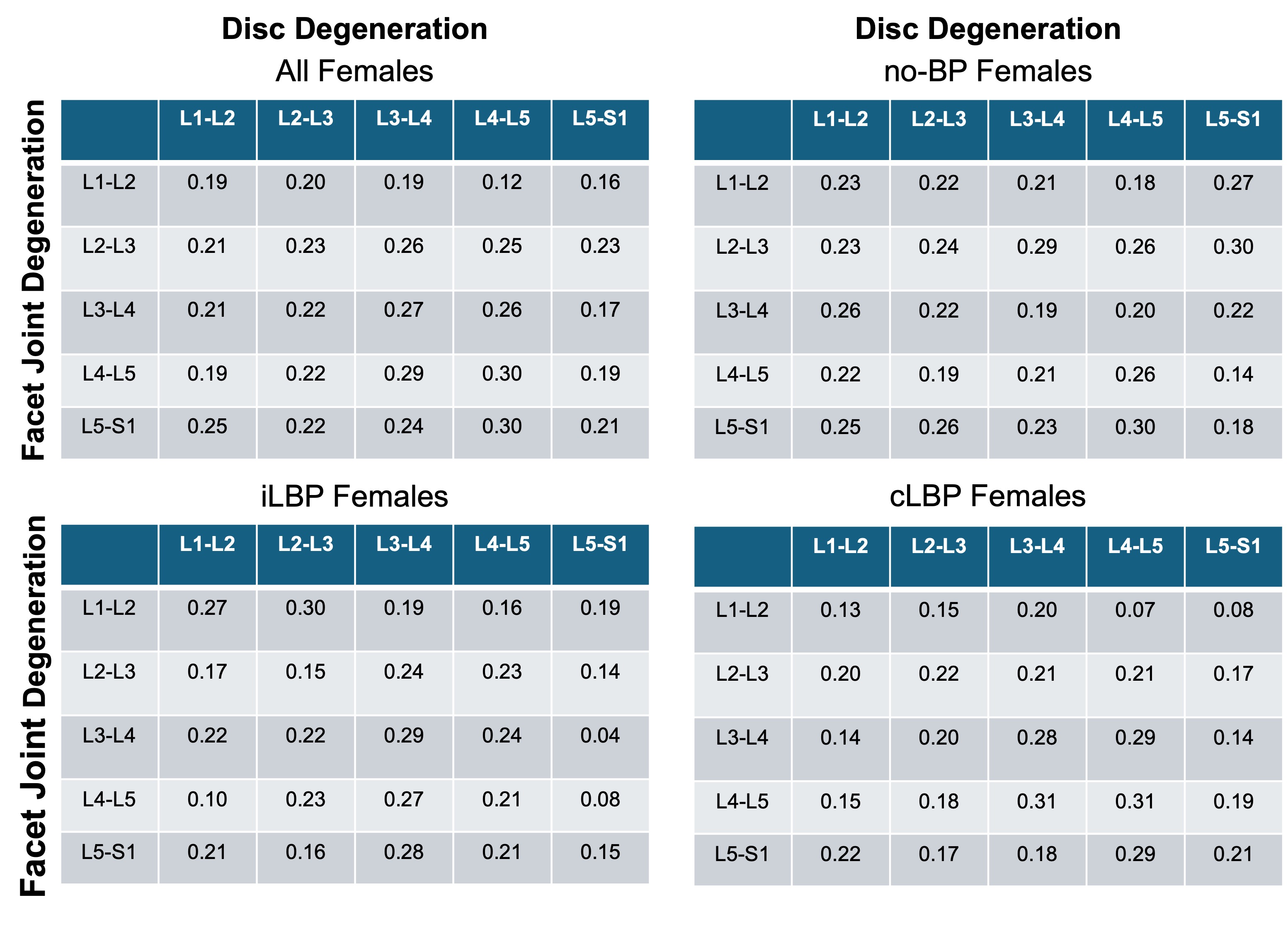

Supplement: Supplementary file 3 [file Image4.jpeg]

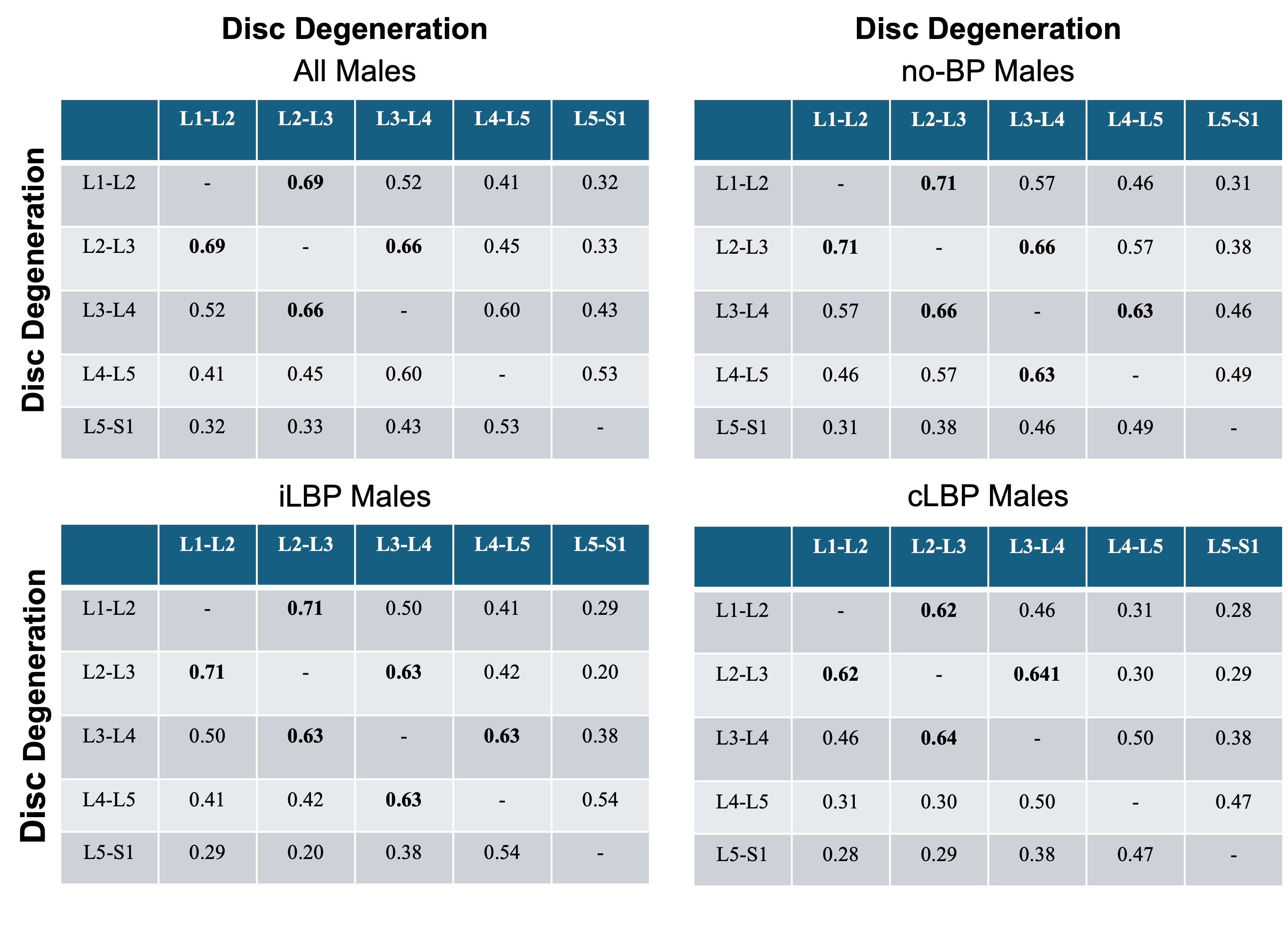

Supplement: Supplementary file 4 [file Image2.jpeg]

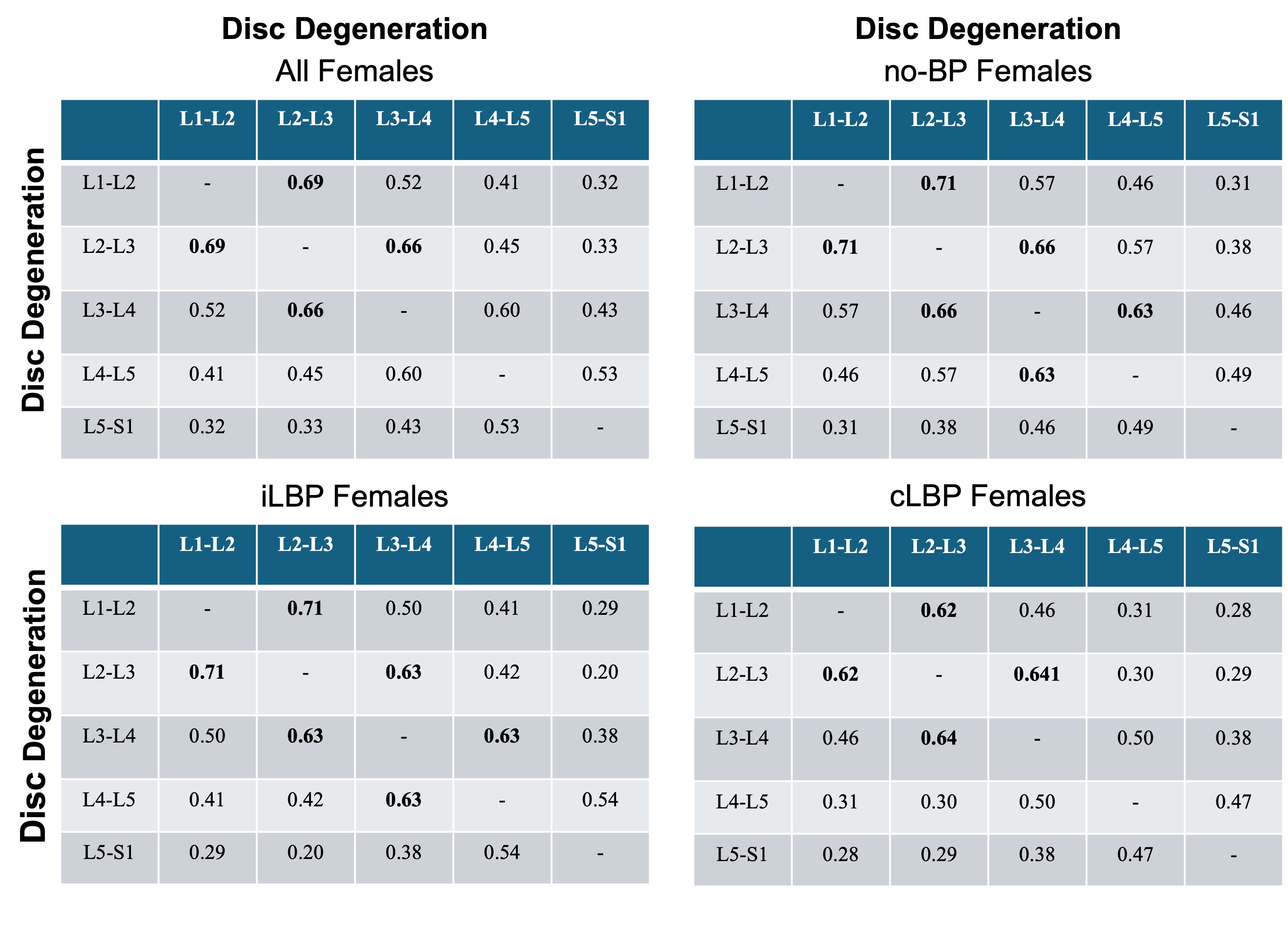

Supplement: Supplementary file 5 [file Image5.jpeg]

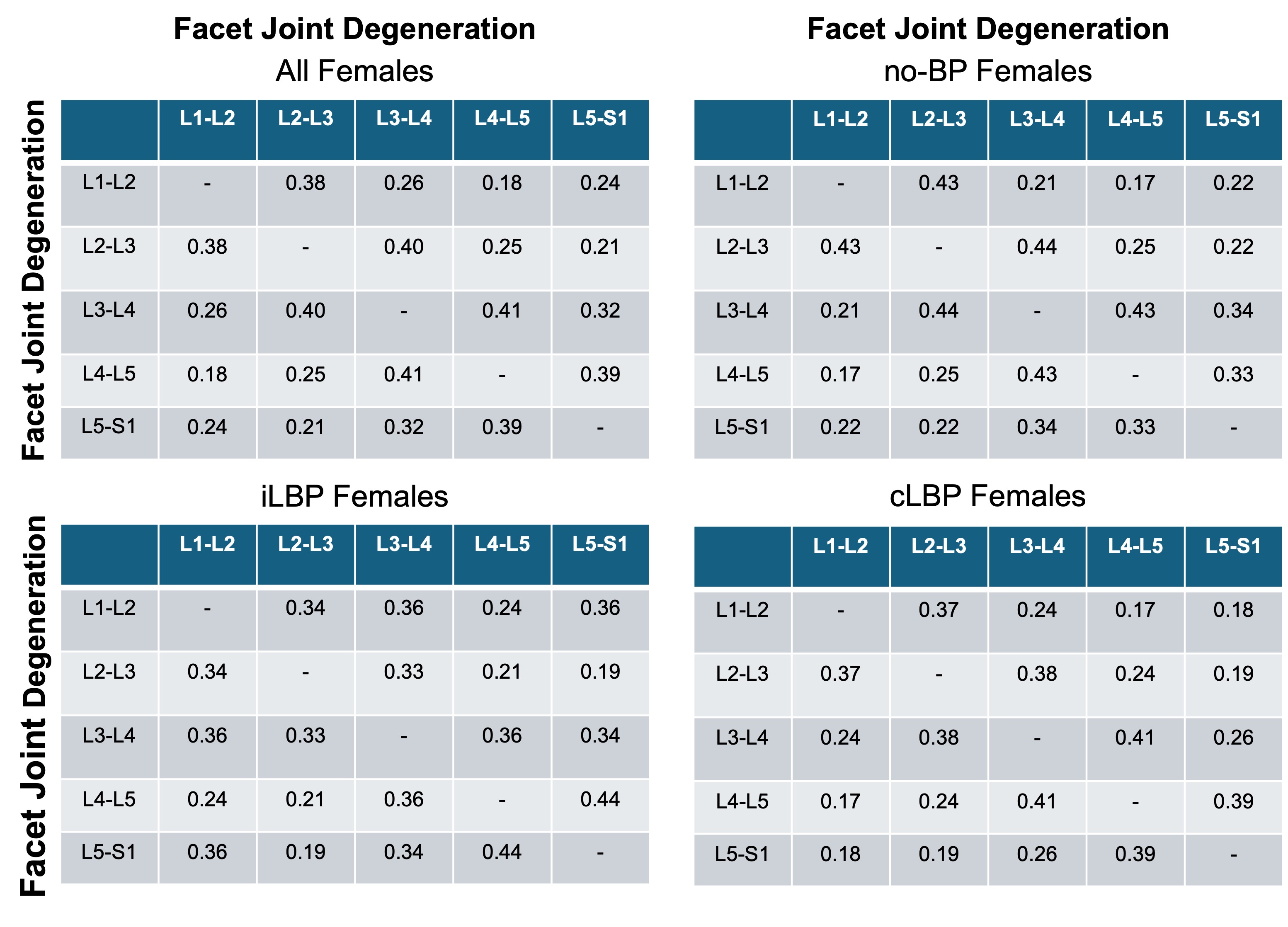

Supplement: Supplementary file 6 [file Image6.jpeg]
